# NLP-enriched social determinants of health improve prediction of suicide death among the Veterans

**DOI:** 10.21203/rs.3.rs-5067562/v1

**Published:** 2025-03-31

**Authors:** Zhichao Yang, Avijit Mitra, Wen Hu, Dan Berlowitz, Hong Yu

## Abstract

Predictions of suicide death of patients discharged from psychiatric hospitals (PDPH) can guide intervention efforts including intensive post-discharge case management programs, designed to reduce suicide risk among high-risk patients. This study aims to determine if additions of social and behavioral determinants of health (SBDH) as predictors could improve the prediction of suicide death of PDPH. We analyzed a cohort of 197,581 US Veterans discharged from 129 VHA psychiatric hospitals across the US between January 1, 2017, and July 1, 2019 with a total of 414,043 discharges. Predictive variables included administrative data and SBDH, the latter derived from unstructured clinical notes via a natural language processing (NLP) system and ICD codes, observed within a 365-day window prior to discharge. We evaluated the impact of SBDH on the predictive performance of two advanced models: an ensemble of traditional machine learning models and a transformer-based deep learning foundation model for electronic health records (TransformEHR). We measured sensitivity, positive predictive value (PPV), and area under the receiver operating characteristic curve (AUROC) overall and by gender. Calibration analysis was also conducted to measure model reliability. TransformEHR with SBDH achieved AUROC of 64.04. Specifically, ICD-based SBDH improved AUROC by 3.1% (95% CI, 1.6% – 4.5%) for the ensemble model and by 2.9% (95% CI, 0.5% – 5.4%) for TransformEHR, compared to models without SBDH. NLP-extracted SBDH further improved the AUROC: 1.7% (95% CI, 0.1%– 3.3%) for ensemble model and 1.8% (95% CI, 0.6%– 2.9%) for TransformEHR. TransformEHR achieved 0.2%, 0.4%, 0.8%, 1.6% PPV per 100 PDPH 7, 30, 90, 180 respectively. Moreover, TransformEHR showed superior calibration and fairness compared to ensemble model, with SBDH further improving fairness across both predictive models. In conclusion, both ICD-based SBDH and NLP-extracted SBDH improved the performance, calibration, and model fairness of prediction of suicide death for Veterans after their psychiatric discharge.

## Introduction

Suicide is a severe and catastrophic phenomenon that deeply impacts individuals, families and communities worldwide. Suicide has remained a grave public health concern for more than a decade. In the US, there were 1.2 million attempted suicides[1] and 45,979 deaths[2] in 2020 alone. Compared to the non-veteran population, Veterans are susceptible to heightened risks associated with suicide. According to the National Veteran Suicide Prevention Annual Report from the US Veteran Health Administration (VHA), the rate of suicide death for Veterans was 1.4 times the rate for non-veteran adults from 2013 through 2019.[3]

Social and behavioral determinants of health (SBDH) refer to the social and behavioral factors that influence an individual's health outcomes and well-being, examples include lack of access to mental health care, unemployment, food insecurity, and exposure to trauma or violence. SBDH can play a crucial role in influencing suicidal behaviors such as ideations and attempts.[4–9] To investigate on more serious outcome such as suicide death, Liu et al. demonstrated a strong correlation between the social vulnerability metric and suicide rates - suicide rates nearly doubled from the least to the most socially vulnerable counties in the United States.[10] Mitra et al. found strong associations between individual

SBDH and suicide death among US VHA Veterans.[11] However, it still remains unanswered whether SBDH can help predict suicide given existing known predictors such as previous suicide attempt.[12,13]

Existing predictive models deployed traditional machine learning ensemble models with predictors including SBDH from structured electronic health records (EHRs).[14,15,16] Structured EHRs, particularly those tailored for billing purposes, frequently exhibit incompleteness when it comes to capturing SBDH.[17,18] Recent research indicates that unstructured EHRs such as notes cover approximately 90 times more SBDH than their structured counterparts.[19] This encouraged us to investigate both SBDH from structured data and SBDH from notes to gauge their roles in predicting suicide. Due to the elevated risks of suicide among patients discharged from psychiatric hospitals (PDPH) and the growing public concern about Veterans' mental health,[14,20] we chose the patient data of Veterans, discharged from VHA psychiatric units for our work.

Building on this foundation, it is imperative to explore how different predictive models, especially those incorporating additional predictors like SBDH, can mitigate potential biases in suicide predictions. Despite recent progress in suicide predictions, substantial challenges remain in addressing demographic disparities.[21,22] Notably, performance discrepancies often occur along demographic lines, particularly with regard to gender[23,24], highlighting the critical need for models that not only enhance prediction accuracy but also fairness. Studies have shown that different genders often respond to social stressors differently.[25,26] To our knowledge, this is the first study evaluating whether SBDH, especially NLP-enriched SBDH enhance model calibration and fairness.

In summary, we aim to answer the following questions in this work:

(1) Can SBDH identified from EHR notes using natural language processing (NLP) further improve suicide prediction?

(2) Are improvements in suicide prediction by SBDH consistent across different machine learning models including an ensemble of traditional machine learning models[14,15] or deep learning generative foundation model[27,28]?

(3) Does the integration of SBDH reduce gender biases and improve model calibration of both traditional and deep learning suicide prediction models?

### Methods

## Cohort

We collected 414,043 short-term (less than 365 days) US VHA psychiatric hospitalizations with discharge dates between January 1, 2017, and July 1, 2019 (Figure 1A). National Death Index (NDI)[29] was used to determine suicide death. Codes used to identify psychiatric hospital discharges are included in Supplementary Table 1. The cases were defined as hospitalizations where patients died in calendar years 2017–2019 and had intentional self-harm (ICD-10: *U03, X60-X84, Y87.0) as the underlying cause of death. This case definition excludes deaths by other causes such as accidents or homicides. The controls were the remaining hospitalizations where patients did not die from suicide.

We observed each hospitalization for 365 days (observation window) prior to the discharge to extract predictors and used those to train multiple machine learning models to predict suicide death within the next 7, 30, 90, and 180 days (prediction window) of that discharge (Figure 1B). We excluded 426 patients who did not have any EHR predictors in their observation windows. We also removed 5 patients with erroneous demographic information. Sociodemographic characteristics of the cohorts are presented in Supplementary Table 2. To measure cross-site generalizability, we evaluated our models on "unseen" test data. Specifically, we stratified the test data based on the discharging hospital and identified data where the discharging hospital differed from the training cohort.

## Predictors

We extracted more than seventy thousand predictors from both structured EHRs (for example: ICD codes) and unstructured EHR notes (for example: hospital admission notes) from the VHA Corporate Data Warehouse (CDW). We also included Area Deprivation Index (ADI)[30], which represents the socioeconomic status of Block Group-level neighborhoods where patients reside. Table 1 lists all categories of predictors.

*Administrative Data*

We collected ICD-10-CM and ICD-10-CPS codes (51,879 unique codes for diagnoses and procedures, excluding ICD-10-CM "Z codes"), Current Procedural Terminology (CPT) codes (10,767 unique codes), and Veterans Health Administration National Drug File (VANDF) codes (1,874 unique codes).

*Demographics and ICD-based SBDH*

We also collected socio-demographic and SBDH (ICD-10-CM "Z codes"). We included 7 socio-demographic predictors: age, sex, race, marital status, income, government job status (whether patient is currently employed by a government agency). Government job status was included as a sociodemographic predictor due to the distinct occupational environments, stressors, and organizational cultures inherent in public sector employment, which can influence mental health outcomes and suicide risk.[31] SBDH were extracted based on ICD-10-CM "Z codes" (Z55-Z65 and Z69-Z99), which were used to identify non-medical factors that may influence a patient's health status, such as the member's socioeconomic situation, including education and literacy, employment, housing, lack of adequate food or water, loss of a family member, etc.[32–34] We refer to this group of SBDH as ICD-based SBDH.

*NLP-extracted SBDH*

To extract SBDH from EHR notes, we deployed an NLP system[11] to identify 9 SBDH predictors from EHR notes, namely, social isolation, transition of care, barriers to care, financial insecurity, housing instability, food insecurity, violence, legal problems, substance abuse.

We also identified two additional predictors from EHR notes, making the total of 11 NLP-extracted predictors. The two predictors are pain, which has been recognized as risk factor of suicide,[35,36] and psychiatric symptoms (e.g., stress, anxiety, and depression), which has been frequently included also as a SBDH domain.[37,38]

For each SBDH predictor, we also identify its presence categories: ‘Current’, ‘History’, ‘No’, ‘Unknown’. This yielded 44 unique SBDH with their associated record dates (11 SBDH predictors X 4 categories). Appendix 1 provides a detailed description of our note selection and presence categories.

*Neighborhood Area Deprivation Index (ADI)*

Our predictors also included neighborhood and community characteristics. Specifically, We linked EHR to the Area Deprivation Index (ADI) data, which is a Census Block Group dataset hosted at Neighborhood Atlas.[30] The ADI data have state-level and national-level rankings of neighborhoods by socioeconomic disadvantage. The index includes factors of income, education, education, employment, and housing quality.[30]

## Statistical Analysis Methods

To test the effect of SBDH on suicide prediction, we chose two different machine learning models (an ensemble of traditional machine learning models[14,15] and TransformEHR[27]). The ensemble method consists of multiple models: logistic regression, gradient boosting[39], extra trees[40] and support vector machine[41]). The TransformEHR model used in this study is designed to process longitudinal events. Specifically, it captures temporal dynamics by sequentially modeling EHR predictors over time, enabling tracking and incorporation of changes in patients' SBDH status across multiple time points. To ensure a fair comparison, the same feature transformation techniques, L2 regularization, and hyperparameter tuning strategies were adopted. Appendix 1 provides more training details. Since suicide death is a rare event, our cohort is extremely imbalanced (death ratio: 0.15%). To mitigate the data imbalance challenge, we applied cost-sensitive learning where each sample was weighted by inverse square root of class frequency. This has been shown to be effective against extreme imbalance.[42,43]

We used the area under the receiver operating characteristic curve (AUROC) to measure the general performance of our model. A 2-sided t test was used to determine the significance of AUROC between different models and factors. All significance tests were evaluated at $\alpha = .05$. We also used sensitivity (SN) at threshold K to measure the recall of the models. In clinical setting, this means that if an intensive post-discharge case management program based on the system-in-evaluation were delivered to the top

K% of hospitalized patients with the highest predicted suicide risk, it would capture SN% of the patients who would otherwise die from suicide. We reported positive predictive value (PPV) to measure model's precision. It indicates the fraction of model predicted suicides that were correct. We also reported adjusted PPV, which represents the anticipated proportion of suicides in relation to the total person-years of intervention throughout that specific time frame. In addition, we conducted calibration analysis using expected calibration error (ECE)[44] to measure model's reliability.

All methods described above were performed in accordance with the recommendations laid out in the World Medical Association Declaration of Helsinki and VHA Privacy and Information Security guidelines. The study protocol was approved by the Institutional Review Board at the VHA Bedford Healthcare System under the waiver of informed consent. Data was de-identified and the study was exempted because the research involves only information collection and analysis involving the investigator's use of identifiable information when that use is regulated under 45 Code of Federal Regulations (CFR) parts 160 and 164, subparts A and E, for the purposes of health care operations or research as those terms are defined at 45 CFR 164.512(b).

### Results

## Outcome Distribution

Of the 197,581 unique participants, 561 died from suicide within 180 days after discharge. These 561 Veterans had a total of 629 psychiatric hospitalizations (cases). The remaining patients had a total of 413,414 hospitalizations (controls). We trained predictive models on 287,243 psychiatric discharges (training data, 70% of the total discharges) from January 1, 2017, to August 10, 2018, and conducted evaluation on 126,800 discharges (test data, remaining 30% discharges) from August 11, 2018, to July 1, 2019. The observed suicide rate at the level of hospitalization within 180 days after hospital discharge was 284.5 per 100,000 person-years in the training set (Figure 2A) and 361.4 in the test set (Figure 2B). In both samples, suicide rate has an inverse relationship with time since discharge, with the highest suicide rate among PDPH 7 (890.7–1194.7) and the lowest in the PDPH 90-180 (111.7–140.9) per 100,000 person-years.

## Overall Prediction Results

For prediction of suicide death among PDPH 180, we found that adding ICD-based SBDH improved AUROC by 3.1% (95% CI, 1.6% – 4.5%, $P$ = 0.001) for the ensemble model and 2.9% (95% CI, 0.5% – 5.4%, $P$ = .03) for TransformEHR, compared to models with administrative data alone. Adding NLP-extracted SBDH as predictors further improved AUROC by 1.7% (95% CI, 0.1%– 3.3%, $P$ = .04) for the ensemble model and 1.8% (95% CI, 0.6%– 2.9%, $P$ = .009) for TransformEHR. Adding NLP-extracted psychiatric symptoms and pain as additional predictors improved AUROC further by 0.4% (95% CI, -0.8%– 1.6%, $P$ = .51) for the ensemble model and 0.1% (95% CI, -1.0%– 1.3%, $P$ = .82) for TransformEHR. In contrast, the results of including ADI as part of predictor are mixed. When SBDH was not included as predictors, ADI improved the performance by 2.7% (95% CI, 0.6%– 4.8%, *P* = .02); and when SBDH were included as predictors, the value of ADI diminished. Appendix 1 provides an importance analysis of different predictors. In addition, TransformEHR with SBDH shows improved calibration compared to TransformEHR without SBDH, as shown in Supplementary Figure 1. Specifically, the ECE of TransformEHR with SBDH is 0.014, whereas the ECE of TransformEHR without SBDH is 0.017.

As shown in Table 2, when compared to ensemble of models, TransformEHR achieved better AUROC by 9.0% (95% CI, 7.6% – 10.4%, *P* < 0.001). TransformEHR is also better calibrated than ensemble of models as shown in Supplementary Figure 1. The ECE of TransformEHR is 0.014, while the ECE of ensemble of models is 0.025. This result indicates higher reliability. The AUROC is consistent among different hospitals, even for those with no patients in the training data, as shown in Supplementary Figure 2. Our analysis suggested that the best model was TransformEHR, and the best predictor combination consisted of administrative data, ICD-based SBDH, and NLP-extracted SBDH. We applied this combination to further explore TransformEHR's operating characteristics for suicide outcome prediction among PDPH 7, 30, 90, and 180.

## Operating Characteristics for Suicide

We calculated sensitivity (SN) and positive predictive value (PPV) at different thresholds for our best model and predictor combination. Thresholds included 5%, 10%, and 20% of observations with highest predicted probabilities for suicide based on the model. The SN at 5% threshold were 41.3%, 36.7%, 34.0%, and 45.3% for PDPH 7, 30, 90, and 180, respectively (Table 3). This means that if an intensive post-discharge case management program based on the model were delivered only to the 5% of hospitalized patients with the highest predicted suicide risk, it would capture 34.0%–45.3% of the patients who would otherwise die by suicide. The same program delivered to the 10% and 20% of patients with the highest predicted risk would capture 56.5%–79.4% (10% decision threshold) and 79.4%–91.1% (20% decision threshold) of the patients who would otherwise die by suicide.

The proportion of patients receiving the intervention who would otherwise die by suicide (i.e., PPV) is an important consideration in determining the potential value of any targeted suicide prevention system. PPV increased as the number of patients above the decision threshold decreased, and the time horizon increased. The highest PPV of our model was 1.6% for the 5% suicide death threshold over the 180-day prediction window. In other words, this is the proportion of patients above that threshold who would die by suicide in the 180 days after hospital discharge in the absence of any interventions beyond those currently provided by VHA. By far the lowest PPVs were for the 7-day prediction window, where values were in the range of 0.09%–0.19% across different thresholds.

## Performance among Different Genders

As shown in Supplementary Table 2, our cohort is male predominant (92%). Therefore, it is important to measure model fairness by gender. Supplementary Figure 3 presents the AUROC of two predictive models (TransformEHR and an ensemble model) by gender. Specifically, TransformEHR model improved AUROC to 61.6 (95% CI, 6.9% – 10.7%, *P* < .001) for males and 55.6 (95% CI, 8.5% –16.4%, *P* < .001) for females. Therefore, TransformEHR outperformed the ensemble model for both male and female. Importantly, the performance gap between male and female was narrower in TransformEHR (9.5%) than in the ensemble model (11.9%), indicating that TransformEHR is a better model for fairness.

We evaluated the impact of SBDH on model performance and fairness. As shown in Supplementary Figure 3, the ensemble model, utilizing only administrative data, shows an AUROC of 56.6 for male and 49.4 for female. When SBDH were added, the performance improved to 59.2 (95% CI, 2.7% – 6.7%, *P* < .001) for male and 52.5 (95% CI, 2.6% –10.2%, *P* = .005) for female. Moreover, the improvement for female was larger: 6.3% for female and 4.7% for male, indicating the improvement of both model performance and fairness by SBDH.

Similarly, adding SBDH improved both model performance and model fairness of TransformEHR. Incorporating SBDH to the TransformEHR model improved AUROC to 64.5 (95% CI, 3.6% – 6.0%, *P* < .001) for males and 58.6 for females (95% CI, 3.4% – 7.6%, *P* < .001) and the improvement is higher for females (5.9%) than for males (4.8%), bringing in the performance closer by gender.

## Discussion

In this study, we examined the importance of NLP-extracted SBDH for suicide prediction. We demonstrated that both ICD-based SBDH and NLP-extracted SBDH can significantly improve a predictive model’s performance. Compared to models without SBDH, model with structured and NLP-extracted SBDH improved AUROC by 4.8% (95% CI, 3.7% – 5.9%, *P* < .001) points for the ensemble of models and 4.7% (95% CI, 2.3% – 7.2%, *P* = .002) points for TransformEHR. Our findings differ from previous research that found that ICD-based SBDH from 40 ICD-9 V-codes have negligible effect on suicide prediction among primary care.[13] However, other studies reported significant associations between SBDH and suicide death. For example, the study by Blosnich et al. used 7 types of SBDH from more than 100 ICD-10 Z-codes and showed that SBDH were highly related to suicide even after adjusting for previous mental health diagnoses.[32] More importantly, they found that each additional SBDH increased the odds of suicide by 49%. Mitra et al. showed that both NLP-extracted social determinants of health (SDOH) and structured SDOH are closely associated with Veterans’ suicide death before year 2015.[11,45] However, these studies focused on associations whereas we emphasized the roles of SBDH on suicide prediction after year 2016.

Across the 4 prediction windows (7, 30, 90, 180 days), TransformEHR with SBDH achieved 0.2%, 0.4%, 0.8%, 1.6% PPV per 100 patients; 10.4%, 4.9%, 3.2%, 3.1% adjusted PPV per 100 patient-years; and 41.3%, 36.7%, 34.0%, 45.3% SN at a specificity of 95%. According to an earlier work, to be cost-effective from a health care management perspective at a specificity of 95%, a suicide screening method would need to have at least a sensitivity of 35.7% and PPV of 0.2% to target cognitive behavioral therapy (CBT) intervention. [46] Other researchers might argue that low PPV in our model would mean that interventions focused on patients classified as high risk would “subject many patients, who will never die by suicide, to excessive intrusion or coercion”. [47] However, such tools still continue to be widely used in clinical practice settings. [48] This could be explained by 2 reasons. First, our method only requires EHRs to be collected passively, compared to extensive questionnaires and clinical psychosocial sessions with expensive medical expert interaction. [49] If such method is deployed on large cloud computing services like Amazon Web Service, the estimated cost will be about $1 per 15,000 candidates for daily screening (as of 05/25/2024). Second, such suicide death screening tool also finds other patients who suffers from severe psychiatric symptoms. Even though the PPV of suicide death was 0.3% for suicide death prediction among PDPH 30 at 90% specificity, the PPVs of suicide attempt, suicide ideation, and major depressive disorder were much higher at 2.0%, 33.2%, and 43.4% respectively among PDPH 30. Intensive post-discharge case management program applied to these patients would help them improve their psychiatric conditions as well. We also found that our model achieved 9.9% PPV and 33.3% SN at a specificity of 95% among suicide death patients who previously had suicide attempt. This PPV is much higher than PPV of suicide death (1.6%). Both exceeded the PPV threshold for CBT intervention among primary care patients (0.2%).[46]

Our results, as shown in Supplementary Fig. 3, demonstrate that TransformEHR not only outperformed the ensemble model in AUROC, but also performed better in gender fairness. We believe that the performance gain may be due to improved feature representation by pretraining where TransformEHR creates a holistic latent feature representation by learning from longitudinal EHRs of millions of patients. Such deep feature representations may capture subtle variations and patterns overlooked by traditional models.[50]

Previous research show that different genders often respond to social stressors differently,[25] and that the presence of recent stressors and participation in social activities increased the odds of suicide among males, while younger age and disrupted family relationships were important predictors among females.[51] In alignment with the aforementioned research, our results, as described in Supplementary Fig. 3, show that incorporating SBDH as additional features not only improved the performance but also fairness for both TransformEHR and the ensemble model, demonstrating the importance of SBDH and NLP-enriched SBDH for prediction of suicide.

## Limitations

First, the study was conducted in VHA cohort where most patients were male. This imbalance elevates the risk of gender-related biases, making gender the primary focus for fairness assessment. Consequently, the evaluation of fairness across other demographic dimensions, such as race or socioeconomic status, was limited and remains an important area for future direction. Second, the study did not include other VHA high-risk patient populations including psychiatric outpatients after recent visits[52], or emergency department patients who need a suicide risk assessment[53]. Third, NLP-extracted SBDH resulted in limited improvement in performance compared to the ICD-based SBDH. Adverse SBDHs are linked to negative health outcomes, including mental health conditions such as depression.[54] These health outcomes are documented using ICD codes, which act as mediators, thereby reducing the direct attribution of SDOH to suicide in clinical records. Fourth, we utilized conventional natural language processing (NLP) techniques to extract SBDH from 4646 clinical notes from real world patients. We did not generate synthetic notes by advanced large language models (LLMs).[55] This is important because integrating synthetic data from LLMs can enhance the NLP model's ability to recognize diverse expressions of SBDH, potentially improving the accuracy and generalizability of the extracted information. Finally, the NLP model had limited F1 score of 0.78 in extracting SBDH. Incorrect NLP-extracted SBDH may further result in lower accuracy in suicide prediction. To account for measurement errors in SBDH, future work could apply Errors-in-variables methods.[56]

### Conclusions

Our findings reveal that SBDH improves prediction of suicide among PDPH. While ICD-based SBDH consistently improved prediction across different models over a broad range of prediction windows, NLP-extracted SBDH further improved the prediction. Our results support the importance of leveraging risk factors from clinical notes for suicide prediction.

### Declarations

## Author Contribution

H.Y. initialized the conceptualization of the project. Z.Y. designed the study, implemented the methods, and performed the data analysis. H.W. checked the validity of the data. Z.Y and A.M. interpretate the results with substantial input from D.B. and H.Y. All authors contributed to manuscript preparation.

## Acknowledgement

Support for VA data was provided by the VA Health Services Research and Development Service. This work was supported by the grant R01MH125027 from the National Institute of Mental Health (NIMH) of the National Institutes of Health (NIH). The contents of this paper do not represent the views of NIH, VA, or the United States Government.

## Data Availability

The datasets generated and/or analysed during the current study are not publicly available due to U.S. VHA Directive 1605.01 and Confidentiality of Certain Medical Records 38 U.S.C. § 7332, but are available from the corresponding author on reasonable request.

## Code Availability

Our code is attached in zip files and will be made publicly available when accepted. Experiments were conducted using Python version 3.8, torch version 1.9.0, transformer library version 4.16.2.

## Tables

Table 1. Predictors used in this experiment.

| Names | Categories | Descriptions | Sources |
|---|---|---|---|
| Administrative Data | Prior suicide attempt | T14.91 and other intentional self-harm like poisoning. | Structured EHRs |
| | Current suicide ideation | R45.85. | |
| | Psychiatric conditions | ICD-10-CM codes included in categories F01-F99 such as Major Depressive Disorder, Schizophrenia, Bipolar Disorder, Post-traumatic Stress Disorder, Substance Use Disorders [42]. | |
| | Chronic pain from physical disorder | Chronic pain is a common risk factor for suicide [43–45]. This includes ICD-10-CM codes related to back pain, neck pain, joint pain and arthritic disorders, abdominal and bowel pain and headache. | |
| | Medical procedures related to psychiatric conditions | ICD-10 PCS and CPT codes including group psychotherapy, family psychotherapy, acupuncture. | |
| | Medications thought to cause suicide | VANDF codes including lorazepam, propranolol, trihexyphenidyl, fluphenazine identify suicide related drugs that can trigger side effects of antipsychotics that often contribute to suicide attempts [46–48]. | |
| ICD-based Social and Behavioral Determinants of Health (SBDH) | | ICD-10-CM “Z codes”. | |
| NLP-extracted SBDH | | Current, History, No and Unknown of each SBDH predictors. | Unstructured Notes |
| Area Deprivation Index | | Both state and country level ADI ranking of patients living neighborhood was used. | Census/Survey |

Table 2. Area under the receiver operating characteristic curve (AUROC) for suicide death prediction within 180 days after psychiatric discharges. Base includes administrative data (ICD, CPT, VANDF codes). SBDH1 refers to ICD-based SBDH. SBDH2 refers to NLP-extracted SBDH. SBDH3 refers to NLP-extracted psychiatric symptoms and pain.

| Models | Factors | AUROC | |
|---|---|---|---|
| | | Mean % | st.dev % |
| Ensemble | Base | 56.0 | 0.3 |
| | Base+Demographics | 56.7 | 0.5 |
| | Base+Demographics+ADI | 58.5 | 0.4 |
| | Base+Demographics+SBDH1 | 57.5 | 0.7 |
| | Base+Demographics+SBDH1+SBDH2 | 58.5 | 0.5 |
| | Base+Demographics+SBDH1+SBDH2+SBDH3 | **58.7** | 0.5 |
| | Base+Demographics+SBDH1+SBDH2+SBDH3+ADI | 58.4 | 0.6 |
| TransformEHR | Base | 61.1 | 1.4 |
| | Base+Demographics | 62.1 | 1.1 |
| | Base+Demographics+ADI | 63.7 | 0.6 |
| | Base+Demographics+SBDH1 | 62.9 | 0.6 |
| | Base+Demographics+SBDH1+SBDH2 | 63.9 | 0.5 |
| | Base+Demographics+SBDH1+SBDH2+SBDH3 | **64.0** | 0.6 |
| | Base+Demographics+SBDH1+SBDH2+SBDH3+ADI | 63.8 | 0.9 |

Table 3. Operating characteristics at a range of thresholds of the transformer trained with training set and applied in the test set to predict suicides death over each of the 4 time horizons (7, 30, 90, 180 days).

| Threshold P | PPV | | SN | |
|---|---|---|---|---|
| 7 days | % | st.dev | % | st.dev |
| 0.05 | 0.189 | 0.011 | 41.287 | 5.861 |
| 0.10 | 0.150 | 0.047 | 65.034 | 4.877 |
| 0.20 | 0.091 | 0.005 | 79.401 | 3.095 |
| 30 days | | | | |
| 0.05 | 0.388 | 0.053 | 36.716 | 1.878 |
| 0.10 | 0.299 | 0.084 | 56.485 | 1.386 |
| 0.20 | 0.220 | 0.067 | 83.080 | 1.996 |
| 90 days | | | | |
| 0.05 | 0.819 | 0.094 | 33.971 | 1.615 |
| 0.10 | 0.730 | 0.166 | 75.874 | 1.148 |
| 0.20 | 0.500 | 0.118 | 91.148 | 1.980 |
| 180 days | | | | |
| 0.05 | 1.632 | 0.332 | 45.300 | 1.222 |
| 0.10 | 1.428 | 0.407 | 79.440 | 1.440 |
| 0.20 | 0.812 | 0.118 | 91.103 | 1.818 |

## Figures

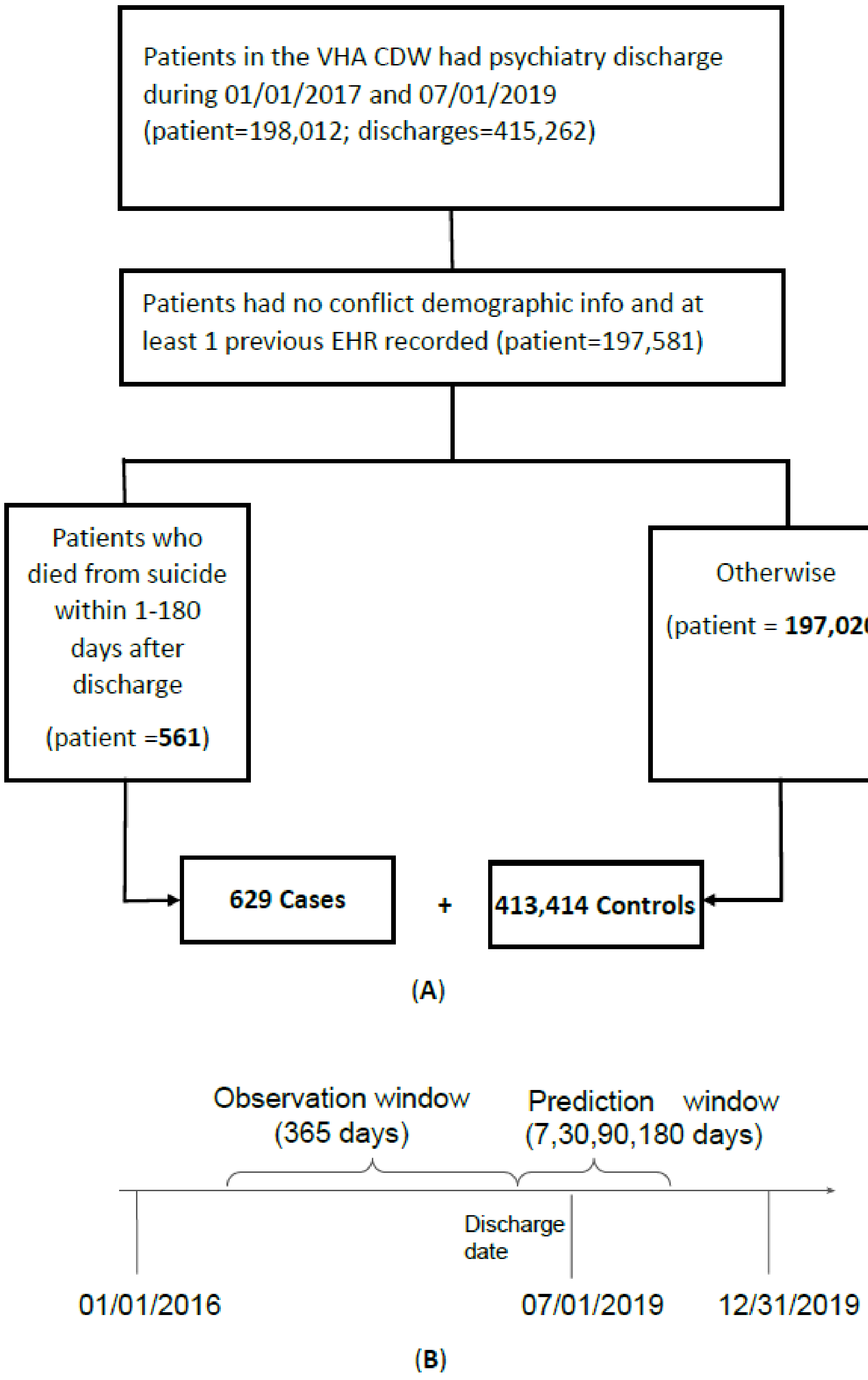

**Figure 1**

(**A**) Cohort definition for suicide death prediction and (**B**) our study timeline.

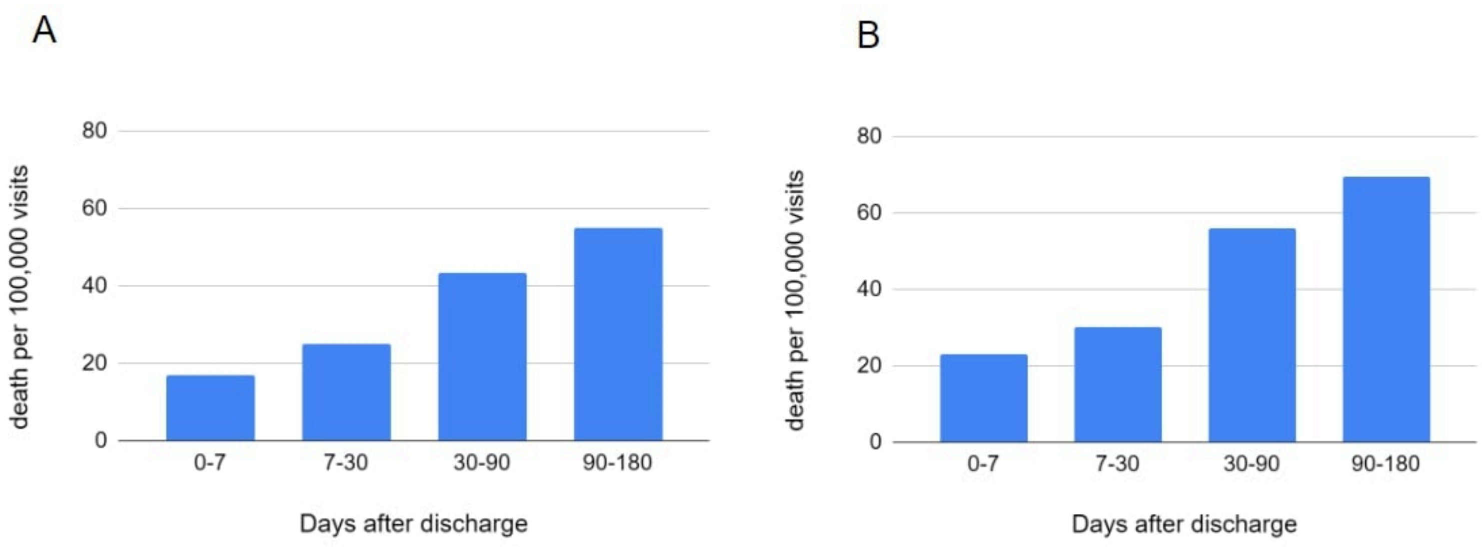

**Figure 2**

Suicide death rates over the 180 days after psychiatric hospital discharge in (**A**) training set (January 1, 2017–August 10, 2018) and (**B**) test set (August 11, 2018–July 1, 2019).

## Supplementary Files

This is a list of supplementary files associated with this preprint. Click to download.

- code.zip
- supplement.docx
